# SMARTPOP: inferring the impact of social dynamics on genetic diversity through high speed simulations

**DOI:** 10.1186/1471-2105-15-175

**Published:** 2014-06-09

**Authors:** Elsa G Guillot, Murray P Cox

**Affiliations:** 1 Statistics and Bioinformatics Group, Institute of Fundamental Sciences, Massey University Palmerston North, New Zealand

**Keywords:** Population genetics, Mating systems, Forward-in-time simulation

## Abstract

**Background:**

Social behavior has long been known to influence patterns of genetic diversity, but the effect of social processes on population genetics remains poorly quantified – partly due to limited community-level genetic sampling (which is increasingly being remedied), and partly to a lack of fast simulation software to jointly model genetic evolution and complex social behavior, such as marriage rules.

**Results:**

To fill this gap, we have developed SMARTPOP – a fast, forward-in-time genetic simulator – to facilitate large-scale statistical inference on interactions between social factors, such as mating systems, and population genetic diversity. By simultaneously modeling genetic inheritance and dynamic social processes at the level of the individual, SMARTPOP can simulate a wide range of genetic systems (autosomal, X-linked, Y chromosomal and mitochondrial DNA) under a range of mating systems and demographic models. Specifically designed to enable resource-intensive statistical inference tasks, such as Approximate Bayesian Computation, SMARTPOP has been coded in C++ and is heavily optimized for speed and reduced memory usage.

**Conclusion:**

SMARTPOP rapidly simulates population genetic data under a wide range of demographic scenarios and social behaviors, thus allowing quantitative analyses to address complex socio-ecological questions.

## Background

Often studied in isolation, interest is now increasingly focused on how non-genetic factors, such as social behaviors, influence population genetic diversity. The pioneering social anthropologist Claude Lévi-Strauss [1] exhaustively described global variation in human marriage systems, and population geneticists are now beginning to explore how marriage rules affect patterns of human genetic diversity [2,3]. Because societies typically dictate different rules for men and women, genetic loci on the sex-linked X and Y chromosomes, as well as mitochondrial DNA (mtDNA), often respond in different ways. The impact of some social processes has been explored analytically [4,5], but the inherent complexity of genetic and social systems limits mathematical results to relatively simple questions.

Limited progress in this field can in part be attributed to a paucity of appropriate simulation tools. Coalescent theory, the workhorse of modern population genetics, makes the strict assumption of random mating (a necessary condition of ‘exchangeability’). Because marriage rules automatically impose non-random mate choices, coalescent approaches (and other simulation programs that make this assumption) cannot be employed. Some forward-in-time simulators do possess the required flexibility to accommodate complex social rules – simuPOP being an excellent example [6]. However, this application is written in the interpreted language Python, and the price of its flexibility is markedly reduced speed (see Table 1). Other software, such as Fregene [7], are fast but cannot simulate sex-specific genetic loci or mating alliances. Modern statistical inference procedures, such as Approximate Bayesian Computation (ABC), are extremely resource intensive, and demand simulation tools that can perform at least an order of magnitude faster than most current applications. SMARTPOP, written in parallelized C++ code and heavily optimized for speed and reduced memory usage, is designed to fit this niche.

**Table 1:** Runtime benchmarking (in seconds) against comparable forward-in-time population genetic simulators

**Population size**	**500**				**1,000**			
**Length of the DNA locus**	**500**		**1,000**		**500**		**1,000**	
**Number of generations**	**1,000**	**10,000**	**1,000**	**10,000**	**1,000**	**10,000**	**1,000**	**10,000**
**SMARTPOP**	**11**	**102**	**14**	**140**	**13**	**130**	**30**	**290**
simuPOP [6]	75	896	134	1,640	121	1,510	260	2,930
NEMO [8]	960	9,390	1,790	17,800	1,990	18,950	3,870	35,900
quantiNEMO [9]	467	4,870	1,050	10,300	1,630	11,300	3,650	23,700
Fregene [7]	126	2,450	188	3,390	179	7,890	370	9,050
GenomePop [10]	58	562	57	560	114	1,118	112	1,119
SLiM [11]	32	327	33	351	63	681	64	763

## Implementation

SMARTPOP – *S*imulating *M*ating *A*lliances as a *R*eproductive *T*actic for *Pop*ulations – implements a forward-in-time simulation framework. Each individual carries a complete set of DNA, comprising sequences of unlinked loci on the autosomes, X chromosome, Y chromosome and mtDNA, which are inherited in the appropriate biological manner. Populations are defined by the user and evolve forward-in-time. The number of loci and their lengths can be chosen by the user.

Each simulation can be considered as containing three features: 

•A demographic model, such as changes in population size.

•A set of mutation rates for different loci. By default, SMARTPOP implements Kimura’s two-parameter mutation model.

•A set of marriage rules – currently monogamy, polygamy, polygyny, polyandry and close-relative inbreeding avoidance, although a wider range of models are under active development.

The challenge of all forward-in-time simulators is how to define the initial state of the simulation [12,13], as neither extreme condition – all individuals identical or all individuals different – is biologically meaningful. One possibility is to allow the deme to evolve for a sufficiently long time (i.e., well beyond the mean time to the most recent common ancestor), such that starting conditions no longer affect the progression of the simulation. However, this approach is computationally wasteful and assumes that population diversity starts from an equilibrium. As an alternative, we allow an optional buffering phase before each simulation, which employs an elevated mutation rate to reach levels of within-population diversity chosen by the user. This ‘accelerated’ evolutionary process mimics natural patterns of genetic variation (both polymorphisms and haplotypes) generated under standard runs, but with a much reduced runtime (see Additional file 1 for details). From this point, the population evolves for a user-defined number of generations under a set of demographic constraints and marriage rules. To simulate complex social and demographic scenarios, the user can save, stop and restart the simulations with different parameters (e.g., constant population size followed by growth to model a settlement event).

SMARTPOP reports a battery of summary statistics and/or full DNA sequences both at the end of the simulation, and if requested, at set time intervals during the run. Summary statistics include the number of segregating sites *S*, Watterson’s theta *θ*_*w*_, the mean pairwise distance and its related diversity index *θ*_*π*_, the number of haplotypes *h*, allelic heterozygosity *H*_*A*_ and Nei’s mean heterozygosity per site *H*_*N*_. Summary statistics (or DNA sequences) can be returned for the entire deme, or for a user-defined sample (i.e., to mimic population sampling in the real world).

A key feature of SMARTPOP, compared with other forward-in-time simulators, is its speed. Simulating DNA sequences for every individual within a population requires substantial computational resources, and runtime often increases linearly with the length of the locus. Benchmarking against other forward-in-time software shows that SMARTPOP can simulate datasets of a few thousand nucleotides within seconds, whereas alternative simulators may take minutes to hours (see Table 1). SMARTPOP gains its speed from i) a code base written in C++, ii) use of the *Boost* library for random computation and optimized array structures, iii) a DNA representation that packs 32 nucleotides into every 64-bit integer, iv) manipulation of DNA sequences by optimized bit operations, and v) code parallelized under the Message Passing Interface (MPI) framework. For most scenarios representative of real human communities, the resulting runtime is less than one second per simulation – often more than an order of magnitude faster than comparable forward-in-time simulators.

Validation formed an integral part of code development. Detailed discussion of the validation process, including comparisons with coalescent expectations, summary statistic matching and metamorphic testing, is presented in Additional file 1.

SMARTPOP is a dynamic, open source project that aspires to provide an extendable statistical tool base for modeling the effects of social behavior on population genetic diversity. It is released with a supporting website containing exhaustive documentation about the source code and model implementation (http://smartpop.sourceforge.net). The code is under active development to address a range of ongoing anthropological and ecological questions. For instance, population structure and inter-deme migration are currently being implemented to explore mating systems that depend on spousal exchange between communities. Additional features are planned for subsequent implementation.

## Methods

**Figure 1:**
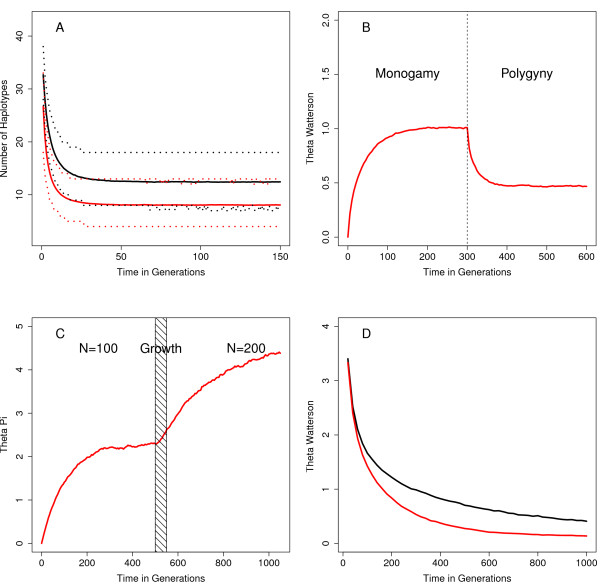
**Four models showing the range of capability of SMARTPOP. A**. Monogamy versus polygyny; **B**. Shift from monogamy to polygyny; **C**. Population growth; **D**. Inbreeding versus sibling avoidance.

To illustrate the range of models that SMARTPOP can simulate, we present four relatively simple case studies (Figure 1).

First, we model genetic diversity on the paternally-inherited Y chromosome through time in two small communities (Figure 1A) – the first monogamous (black), the second polygynous (red). Simulations (*n*=10^4^) modeled 1 Mb of the Y chromosome with a mutation rate of 3×10^−8^ mutations/site/generation in constant sized populations of 200 individuals. Leveraging the buffering phase, we mimic the founding of these two populations from a larger source group with much higher genetic diversity (*θ*_*π*_=25). Figure 1A shows the mean (thick lines) and 95% confidence interval (dotted lines) of the number of Y chromosome haplotypes observed through time.

Second, we model a shift in mating systems. Simulations (*n*=10^4^) modeled 1 Mb of the Y chromosome with a mutation rate of 3×10^−8^ mutations/site/generation in constant sized populations of 100 individuals under a switch from monogamy (generations 0–300) to polygyny (generations 301–600). Figure 1B shows the mean value of Watterson’s theta (*θ*_*w*_) for the Y chromosome through time.

Third, we model genetic diversity in a population experiencing demographic change. Simulations (*n*=10^4^) modeled 1 Mb of the X chromosome with a mutation rate of 3×10^−8^ mutations/site/generation. The population size is initially constant (*n*=100) for 500 generations and reaches an equilibrium state. The population then grows by two individuals per generation for 50 years, after which it evolves for 500 generations with a larger constant size of 200 individuals (consequently reaching a second equilibrium state). Figure 1C shows the mean pairwise diversity (*θ*_*π*_) of the X chromosome through time.

Finally, we model the impact of sibling mating avoidance in small populations. Simulations (*n*=10^4^) modeled 10 fully unlinked autosomal loci, each of 3200 nucleotides, with a mutation rate of 3×10^−8^ mutations/site/generation in constant sized populations of 100 individuals. Leveraging the buffering phase, we mimic the founding of these two populations from a larger source group with higher genetic diversity (*θ*_*π*_=25). Figure 1D shows the mean value of Watterson’s theta (*θ*_*w*_) through time in two polygamous populations that allow (red) or prohibit (black) sibling matings.

## Results and discussion

### Usage

SMARTPOP runs from the command line with user-defined parameter flags. All parameters, except population size, have default values. If desired, parameters can be read from a command file. Given the complexity of the models that SMARTPOP is able to model, the interface is relatively simple and should rapidly become familiar to users of coalescent simulators such as MS [14]. Full documentation and support for using SMARTPOP is available on the project website (http://smartpop.sourceforge.net).

To simulate 500 instances of a 16 kb mtDNA sequence in a population of 200 monogamous individuals (mating system 1), for 100 generations, with a mutation rate of 2×10^−6^ mutations/site/generation, with *θ*_*π*_ (=*N*_*e*_*μ*) reaching 25 in the buffering phase, the following command line would be used:

In the following example, an equivalent set of simulations parallelized under MPI would be distributed across four processors:

### Speed comparison

SMARTPOP has been highly optimized for speed. Simulation runtimes for the serial version of SMARTPOP were benchmarked against comparable forward-in-time simulators. (Note that most of these cannot model social behavior). Table 1 reports runtimes with regard to three main parameters: population size, length of the DNA locus, and number of generations. In all cases, the runtime is reported for 100 simulations of an autosomal locus with a mutation rate of 10^−6^ mutations/site/generation in a constant sized population. The programs were all executed on a Linux system running Ubuntu v. 13.04 with a 3.07 GHz Intel Xeon CPU X5675 processor. Simulations were not memory or I/O constrained. Runtimes for SMARTPOP varied from 2 to 153-fold (mean 41-fold) faster than other software applications (Table 1, time in seconds). The parallel version of SMARTPOP achieves even higher speedup than presented in this benchmarking exercise. Because the Message Passing Interface (MPI) implementation is embarrassingly parallel, runtimes decrease approximately linearly with the number of available cores.

### Worked examples

Figure 1 highlights the large range of scenarios that SMARTPOP is able to model. Figure 1A illustrates the difference in genetic dynamics of small populations following two mating systems, monogamy and polygyny. Both mating systems are found widely in human societies. The population practicing polygyny quickly exhibits lower genetic diversity on the Y chromosome, compared to the monogamous population, due to the higher male variance in number of offspring produced under polygyny [15].

Example 1B explores the effect of a switch in mating systems from monogamy to polygyny. Genetic diversity first reaches an equilibrium under monogamy. After switching to polygyny at 300 generations, genetic diversity decreases to a new equilibrium state of lower diversity. Such shifts between mating systems have also been documented in human communities. A particularly well-known example are the Mormons who practiced polygyny during the early history of the western US [16].

Figure 1C presents the dynamics of genetic diversity following a change in population size. The simulation starts with a constant population size and subsequently reaches equilibrium. The population then doubles over 50 generations. Genetic diversity consequently increases to a new equilibrium point after a significant lag period (here, 200 generations, or approximately 10,000 years). Population growth is a common feature of human populations, particularly during Neolithic expansions [17].

Figure 1D describes an animal mating system with and without inbreeding avoidance. We compare autosomal diversity in small populations that allow or prohibit full and half sibling matings. This scenario formalizes recent observations of chimpanzee inbreeding avoidance, which is assumed to be an evolutionary strategy to increase genetic fitness [18]. These simulations confirm (and quantify) that societies with inbreeding avoidance maintain higher levels of genetic diversity, hence suggesting one possible evolutionary advantage of this practice.

Although these examples are relatively simple for didactic purposes, SMARTPOP can be used to explore far more complex social rules. We emphasize that this software is not specifically designed for humans, and as shown above, can be used to model a much wider range of biological systems in which social behaviors are thought to impact on patterns of genetic diversity.

## Conclusions

Developed to tackle the issue of computational speed when modeling interactions between genetic diversity and social behavior, SMARTPOP simulates complex social and demographic scenarios on a large range of genetic markers (autosomal, X-linked, Y chromosomal and mitochondrial DNA).

The examples presented here illustrate the capacity of SMARTPOP to quantify the impact of social constructs, like mating systems, on population genetic diversity. They also highlight the importance of modeling the dynamics of population genetic diversity through time, emphasizing non-equilibrium outcomes of rapid shifts between social and demographic states over short timescales.

SMARTPOP is well suited for studying human social systems, but is equally applicable to other species that exhibit complex social rules [12,19,20]. SMARTPOP can handle most haploid, diploid or haplo-diploid systems, thus enabling investigation of a wide range of socio-ecological questions in a wide range of social species.

## Availability and requirements

**Project name**: SMARTPOP** Project home page**: http://smartpop.sourceforge.net**Operating system**: Linux, Windows, OS X **Programming language**: C++ **Other requirements**: 64 bit machine; C++ compiler; Boost v. 1.50 or higher**License**: GNU GPL v. 3.0 **Any restrictions to use by non academics**: None

## Competing interests

The authors declare that they have no competing interests.

## Authors’ contributions

EGG designed and developed SMARTPOP, and drafted the manuscript. MPC contributed to software design and analyses, and drafted the manuscript. Both authors read and approved the final manuscript.

## Supplementary Material

Additional file 1 **Implementation and validation.** An extended discussion of implementation choices and a complete description of the software validation process. Click here for file
