# Supplementary material for: SMARTPOP: inferring the impact of social dynamics on genetic diversity through high speed simulations
Source: BMC Bioinformatics. 2014 Jun 9;15:175. doi: 10.1186/1471-2105-15-175 (PMC4064502; doi:10.1186/1471-2105-15-175)
Supplement: Additional file 1 — Implementation and validation. An extended discussion of implementation choices and a complete description of the software validation process. [file 1471-2105-15-175-S1.pdf]

ADDITIONAL FILE 1

# SMARTPOP: Inferring the impact of social dynamics on genetic diversity through high speed simulations

Elsa G Guillot[1] and Murray P Cox[1]

## Validation

### Automatic Test Procedures

Validating deterministic software is straightforward – the same input should always produce the same output. However, because simulators contain random subroutines (e.g., for mating, reproduction and mutation), they are not deterministic and therefore cannot be validated by simple input/output expectations.

The first step towards validation involved testing the complex object-oriented structure of the software. A test suite was implemented with the *Test* library of *Boost* (v. 1.54; `http://www.boost.org`), and a robust and efficient series of automatic tests was developed. These tests, which can be run by end users, check that instances of classes are created correctly, and that deterministic member functions produce expected results (e.g., modification of correct attribute values). Each class in the C++ code is checked independently.

The test suite is available via the commands:

```
> make test
> ./test
```

These test functions have been validated on multiple platforms, including Linux (Ubuntu 13.04, Fedora 17 and Mint 14), Mac OS X (10.8) and Windows (7 and 8).

### Theoretical Expectations

As the output of SMARTPOP is non-deterministic, alternative checks based on mathematical results from theoretical population genetics have been developed to confirm that the system is behaving correctly.

Results from a large number of simulations were compared against values expected under coalescent theory [1]. For example, the mean and variance of the time to the most recent common ancestor (TMRCA) assuming a constant population size [2, 3, 4] is:

$$E(T_{MRCA}) = 2n\left(1 - \frac{1}{n}\right)$$

$$var(T_{MRCA}) = n\left(8\sum_{i=2}^{i=n}\frac{1}{i^2} - 4\left(1 - \frac{1}{n}\right)^2\right)$$

The time to the most recent female common ancestor was simulated for mitochondrial DNA (mtDNA) in a constant sized population with random mating to approximate the Canning's model (i.e., the theory for which the equations above were derived [5]). Figure 1 shows that the mean and variance of 1,000 simulations do not

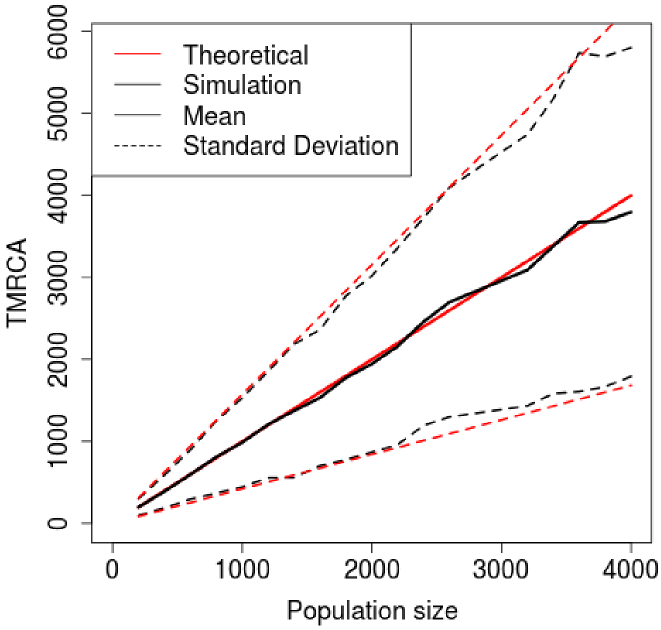


**Figure 1** Time to the most recent (female) common ancestor for mtDNA as a function of the female population size. Red lines show theoretical values; black lines show values simulated by SMARTPOP. Thick lines show the mean; dotted lines show the standard deviation.

vary from theoretical expectations (Student's $t$ test: $P_{\text{mean}} = 0.95$, $P_{\text{variance}} = 0.70$). This test procedure was repeated for both male and female lineages (i.e., mtDNA and Y chromosome) for a range of population sizes.

Second, the simulation model distributes the number of children per woman as a Poisson random variable. We confirmed that simulations produce the correct distribution (i.e., a mean and variance of 2 for constant sized populations).

Third, mating systems were tested by comparing the observed and expected number of mates per individual. Under monogamy, each individual must have no more than one mate. Under polygamy, the mean number of mates must be close to one with some non-zero variance.

## Comparisons with Coalescent Simulators

Coalescent simulators, such as MS [6] and SIMCOAL [7], are used widely in the community to produce simulated population genetics datasets. As such programs reconstruct genetic lineages backward-in-time, they necessarily have strong assumptions (e.g., random mating). To validate our forward-in-time simulator, we compared data simulated by SIMCOAL and SMARTPOP under random mating for defined sets of parameters (e.g., mutation rate and sequence length). To ensure direct comparability, SMARTPOP simulations were first allowed to reach equilibrium by running them for a large number of generations beyond the expected TMRCA.

The two models differ in a second key feature: the backward-in-time process is controlled by the effective population size, while the forward-in-time process is controlled by the census population size. To account for this difference, each SMARTPOP simulation was run under a random census population size, the corresponding effective population size was inferred from the resulting genetic data, and a paired SIMCOAL simulation was

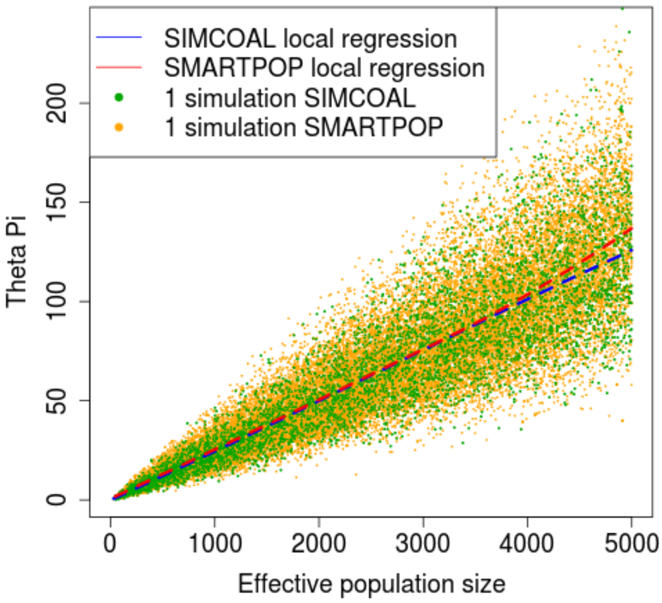


**Figure 2** Pairwise diversity ($\theta_\pi$) of mtDNA in datasets simulated with SMARTPOP (red line and orange points) and SIMCOAL (blue line and green points). Each dot represents a diversity estimate from one simulated dataset. Dashed lines show the local regression to the mean.

run with this value. The mean and variance of several genetic diversity estimators were then compared for both datasets. The two methods produce highly concordant results (Figures 2 and 3).

## Metamorphic Testing

As software has increased in complexity, a new test procedure (metamorphic testing [8]) has been developed to address the problem of validating complex software systems. Within the last few years, metamorphic testing has begun to be applied to bioinformatics software [9, 10]. The approach leverages scaling properties of the simulation model ("metamorphic relations"), for which a defined change in the output can be predicted for a defined change in the input.

The primary challenge is the identification of metamorphic relations appropriate to the problem. Theoretical population genetics suggests several scaling relations. The following cases have been tested in SMARTPOP:

- If the mutation rate is multiplied by a factor $x$, then the diversity estimators $S$, $\theta_w$ and $\theta_\pi$ scale linearly with $x$.
- If the effective population size is multiplied by a factor $x$, then the diversity estimators $S$, $\theta_w$and $\theta_\pi$ scale linearly with $x$.

Because the coalescent comparisons described earlier were performed manually, only a relatively small set of parameters could be tested. Metamorphic testing allows the validation process to be scaled up to a large number of test parameters.

Mean values for 1,000 simulations were tested using a random set of starting parameters (e.g., population size and mutation rate) with $x$ drawn from a random discrete (integer) uniform distribution, $Unif\,(1, 5)$. In all cases, differences between the means of $x \times E(parameter)$ and $E(x \times parameter)$ were less than 10%, thus confirming that the metamorphic relations hold for the simulation software.

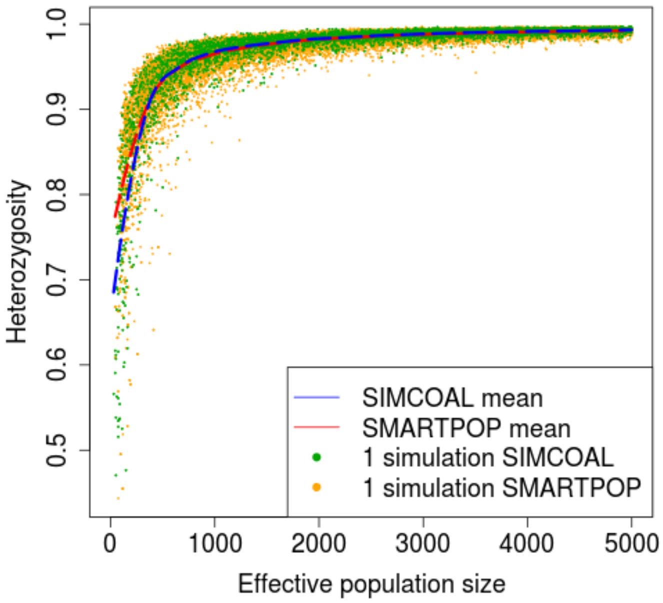


**Figure 3** Allelic heterozygosity ($H_A$) of mtDNA in datasets simulated with SMARTPOP (red line and orange points) and SIMCOAL (blue line and green points). Each dot represents a heterozygosity estimate from one simulated dataset. Dashed lines show the local regression to the mean.

## Summary Comparison

To speed up analyses, several summary statistics are calculated directly within SMARTPOP. To validate these estimators, a series of checks were implemented.

Because most related programs were designed to handle small sample sizes, the population-level dataset simulated by SMARTPOP was sampled randomly. DNA sequences for these simulated individuals were imported into COMPUTE [11] and ARLEQUIN v. 3.5 [12], and the same set of summary statistics returned by SMARTPOP was calculated. The values obtained by SMARTPOP, COMPUTE and ARLEQUIN were then compared across 1,000 simulated datasets (Table 1). Differences in values were negligible – integer summaries were identical; non-integer summaries exhibited extremely low variance due to rounding error. All exceptions ($\theta_w$, $\theta_\pi$ and Tajima's D) result from the implementation of slightly different equations.

| Summary statistics | Formula in SMARTPOP | Comparison with COMPUTE | Comparison with ARLEQUIN |
|---|---|---|---|
| Segregating Sites | $S$ = number of segregating sites | 0 | 0 |
| Haplotypes | $h$ = number of haplotypes | 0 | 0 |
| Heterozygosity ($H_A$) | $H_A = \frac{N}{N-1}\left(1 - \sum_{i=1}^{h} f_i^2\right)$ | $NA$ | $5.1 \times 10^{-5}$ |
| Heterozygosity ($H_N$) | $H_N = \frac{1}{S}\frac{N}{N-1}\sum_{i=1}^{S}\left(1 - \sum_{j=1}^{4} f_j^2\right)$ | $NA$ | $8.5 \times 10^{-4}$ |
| Watterson's Theta | $\theta_w = \frac{S}{\sum_{i=1}^{i=N-1}\frac{1}{i}}$ | $a$ | $1.8 \times 10^{-6}$ |
| Homozygosity Theta | $\theta_H = \frac{1}{(1-H)} - 1$ | $NA$ | $1.4 \times 10^{-3}$ |
| Theta Pi | $\theta_\pi = \frac{N}{N-1}\sum_{i=1}^{h}\sum_{j=1}^{h} dist\,(i,j)$ | $b$ | $4.7 \times 10^{-6}$ |
| Tajima's D | $D = \frac{\theta_\pi - \theta_w}{\sqrt{\left(b_1 - \frac{1}{a_1}\right)\frac{1}{a_1}S + \left(b_2 - \frac{n+2}{a_1 n} + \frac{a_2}{a_1^2}\frac{1}{a_1^2+a_2}\right)S(S-1)}}$ with $a_1 = \sum_{i=1}^{n-1}\frac{1}{i}$, $a_2 = \sum_{i=1}^{n-1}\frac{1}{i^2}$, $b_1 = \frac{n+1}{3(n-1)}$, $b_2 = \frac{2(n^2+n+3)}{9n(n-1)}$ | $c$ | $1.0 \times 10^{-2}$ |

**Table 1** Comparison of summary statistics calculated with SMARTPOP, COMPUTE and ARLEQUIN. The comparison columns show the mean difference in summary values, '$NA$' if the summary is not implemented in the comparison program, or an equation if the implementation differs from that of SMARTPOP.

*a*: Not comparable since the formula implemented in COMPUTE is $\theta_w = \sum_{i=1}^{i=S}\frac{S}{\sum_{j=1}^{n_i-1}\frac{1}{j}}$

*b*: Not comparable since the formula implemented in COMPUTE is $\theta_\pi = \sum_{i=1}^{i=S}\left(1 - \sum_{j=1}^{j=4}\frac{k_{j,i}(1-k_{j,i})}{n_i(n_i-1)}\right)$

*c*: Not comparable as Tajima's D is a function of $\theta_w$ and $\theta_\pi$, both of which differ in COMPUTE.

## Model Implementation

Forward-in-time simulators produce individuals and their DNA sequences using an explicit set of demographic, social and genetic models. While we use models that have wide acceptance in the field, their exact implementation has a direct impact on the simulations. The following sections describe these models in more detail, but much more extensive information is available on the project website (`http://smartpop.sourceforge.net`).

### Demographic Models

Population size can either be constant or change through time, as defined by the user. Population size is controlled internally via the number of offspring. Let $N_t$ be the size of the parent generation. The number of offspring is then calculated using the following demographic function with size change variables $a$, $b$ and $c$ defined by the user:

$$N_{t+1} = a + bN_t + cN_t^2$$

This is a general population size change equation that allows linear, exponential and logistic growth and decline. Once the total size of the next generation is defined, each female (or male in the case of polyandry) is assigned a random number of offspring drawn from a Poisson distribution conditioned on the desired population size. At an individual level, the number of offspring for each female (or male) is a Poisson random variable constrained by the fact that exactly $N_{t+1}$ offspring are born in the population as a whole.

### Social Models

SMARTPOP currently allows several mainstream mating systems to be run.

- Monogamy
  Males and females are paired randomly to mate. No individual can be paired with two or more different mates. The number of offspring per couple is a Poisson random variable.
- Polygamy
  Males and females are paired randomly to mate, with no constraint on mates per individual. The number of mates per individual is a binomial random variable, while the number of offspring per couple is a Poisson random variable.
- Polygyny
  Males and females are paired randomly to mate, with no constraint on mates per male. A female can only mate with one male. The number of mates per male is a binomial random variable, while the number of offspring per female is a Poisson random variable.
- Polyandry
  Males and females are paired randomly to mate, with no constraint on mates per female. A male can only mate with one female. The number of mates per female is a binomial random variable, while the number of offspring per male is a Poisson random variable.
- Random mating
  Males and females are paired randomly to mate, with no constraint on mates per individual. The number of mates per individual is a binomial random variable, while there is no constraint on the number of offspring per individual.

Each mating system contains an option for full and half sibling mating avoidance.

### Defining Starting Conditions

Unlike backward-in-time methods, such as the coalescent, forward-in-time simulations are highly dependent on their starting point. This problem has been raised by other studies [13], but there is little consensus on how to define the initial population. Most programs start from a 'null' population comprising individuals

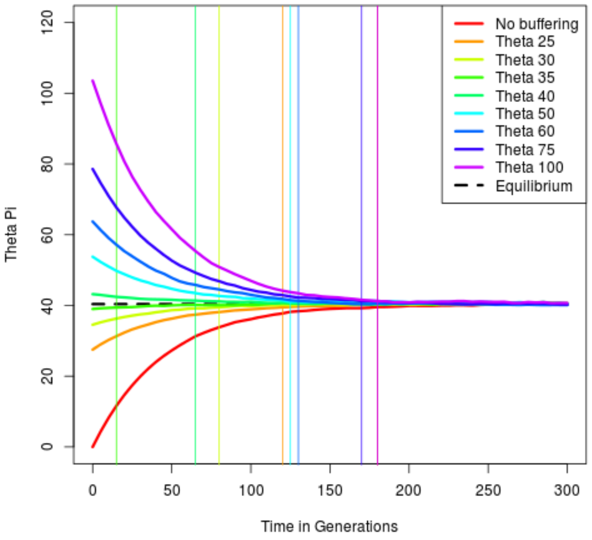


**Figure 4** Simulations across a range of buffering values showing times to equilibrium (vertical colored lines).

that are genetically identical [14]. In such cases, it is typically advised to run the simulations "long enough" (i.e., some long, but undefined period of time) for the system to reach an equilibrium state. This long 'pre-run' stage is often discarded as a burn-in phase, but can require substantial runtime, especially for large populations.

Other programs allow simulations to start from a real population genetic dataset [15], but this requires pre-existing data and is also meaningful only for inferences about the future of a population, not its past.

SMARTPOP provides multiple methods to define a simulation's starting point depending on the user's needs and research questions. By default, a 'null' population of identical individuals is used. This traditional approach is acceptable if users can tolerate long runtimes, and importantly, the assumption of starting from a genetic equilibrium is appropriate for their study system. However, these two assumptions are now critically limiting for many population genetic inference settings.

To speed up simulations, SMARTPOP offers an optional buffering feature. This enacts accelerated evolution using a high mutation rate, which stops after a user-defined diversity threshold is reached. This period of accelerated evolution is then discarded as a burn-in, and the genetic dataset returned by SMARTPOP starts from this point in the run. Buffering is performed independently for each simulation to ensure different random starting points.

Figure 4 explores a range of buffering thresholds to accelerate an example simulation towards its state of equilibrium. Simulations ($n = 10^4$) modeled a 3200 bp sequence of mitochondrial DNA with a mutation rate of $4 \times 10^{-6}$ mutations/site/generation in constant sized monogamous populations of 100 individuals. Mean pairwise divergence ($\theta_\pi$) is plotted through time for each buffering value. Table 2 presents the time in generations taken by each set of simulations to reach equilibrium (defined here as $|\bar{\theta}_\pi(t) - \bar{\theta}_\pi(\infty)| < 1$). The final column

lists the CPU time in seconds to run 100 simulations to equilibrium using the buffering phase. If the buffering threshold is set close to the mean pairwise distance at equilibrium (e.g., $\theta = 35$), the simulation evolves to the equilibrium state faster than if no buffering were used (red). However, if the threshold is far from the equilibrium point (e.g., $\theta = 100$), the simulation can take longer to reach equilibrium. In terms of runtime, simulating this example system with buffering of $\theta = 35$ is twice as fast as starting from the null 'all individuals identical' set. To put this in perspective, optimal buffering could save 1.5 hours of runtime over a standard run of 1,000,000 simulations.

| **Buffering threshold** ($\theta$) | **Time to equilibrium (generations)** | **Runtime (s)** |
|---|---|---|
| No buffering | 180 | 1.02 |
| 25 | 120 | 0.63 |
| 30 | 80 | 0.58 |
| **35** | **15** | **0.46** |
| 40 | 65 | 0.66 |
| 50 | 125 | 0.97 |
| 60 | 130 | 1.14 |
| 75 | 170 | 1.42 |
| 100 | 180 | 1.99 |

**Table 2** Speed gains from buffering.

This discussion raises the issue of equilibrium and its appropriateness for biological modeling. All populations are dynamic – they move, split, merge, grow and contract. Processes that are strongly time localized can have genetic effects over a much longer timeframe (see Figure 1B and 1C of the main article). The modularity of SMARTPOP enables such dynamic studies by saving and reloading simulations with different parameters. This allows users to define any starting point that is the outcome of some prior evolutionary process. For example, it is possible to simulate a population of 100 settlers that recently migrated from a larger population of size 200. One way to do this would be to simulate a population ($n = 200$) until it reaches equilibrium (i.e., a long time), save the simulated populations, and then reload them but this time sampling only 100 individuals. The following command lines show this example:

```
./smartpop -p 200 -t 20 -nstep 50 -sample 50 -sizeMt 3200 -save file1
./smartpop -load file1 -p 100 -t 20 -nstep 50 -sample 50 -o fileresult -mtdiv
```

However, this process is time consuming, especially if it requires a large population to reach equilibrium. Buffering provides an alternative approach. Accelerated evolution can be used to reach a much higher diversity than the equilibrium state, thus mimicking a small population that recently separated from a large one (such as might occur during a settlement event). Figure 5 shows three sets of simulations for a monogamous population of size 100 with the same parameters as the example above, but with different starting points: the null 'all individuals identical' set (black), buffering with $\theta = 75$ (red) and down-sampling as described above (blue). The null 'all individuals identical' method cannot be used to model a settlement event, and is shown here solely to emphasize that all simulations eventually reach the same equilibrium point. Note, however, that buffering creates a diversity dynamic that is concordant with the sampling method, but buffering is much faster (1.03 vs 2.36 s).

These simple examples illustrate the speed gain that buffering can provide for different scenarios. As the simulated population size increases, this gain becomes even more pronounced and buffering may become necessary to keep runtimes to an acceptable level.

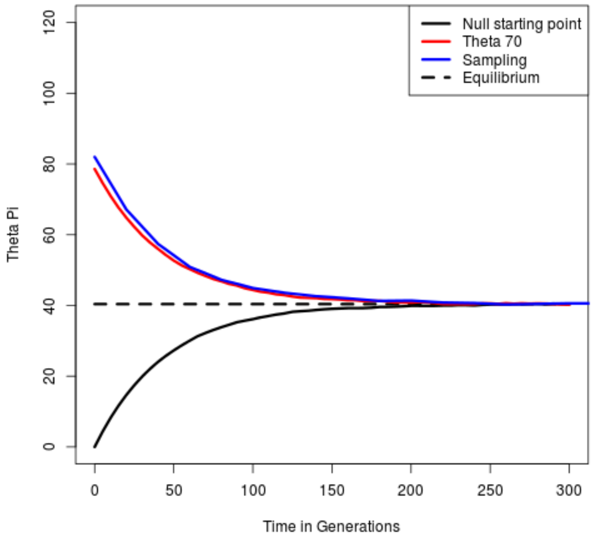


**Figure 5** Simulation of the genetic dynamics of 100 individuals who split from a population of 200 individuals with buffering (red) and sampling (blue) methods.

**Author details**
[1]Statistics and Bioinformatics Group, Institute of Fundamental Sciences, Massey University, Palmerston North, New Zealand. [2].

**References**
1. Kingman, J.F.C.: The coalescent. Stochastic Processes and their Applications **13**, 235–248 (1982)
2. Hudson, R.R.: Gene genealogies and the coalescent process. Oxford Surveys in Evolutionary Biology **7**, 44 (1990)
3. Donnelly, P., Tavaré, S.: Coalescents and genealogical structure under neutrality. Annual Review of Genetics **29**, 401–421 (1995)
4. Wakeley, J.: Coalescent Theory: An Introduction. Roberts & Company Publishers, Greenwood Village, Colorado (2009)
5. Cannings, C.: The latent roots of certain Markov chains arising in genetics: A new approach, I. Haploid models. Advances in Applied Probability **6**, 260–290 (1974)
6. Hudson, R.R.: Generating samples under a Wright-Fisher neutral model of genetic variation. Bioinformatics **18**, 337–338 (2002)
7. Laval, G., Excoffier, L.: SIMCOAL 2.0: A program to simulate genomic diversity over large recombining regions in a subdivided population with a complex history. Bioinformatics **20**, 2485–2487 (2004)
8. Chen, T.Y., Tse, T.H., Zhou, Z.: Semi-proving: An integrated method based on global symbolic evaluation and metamorphic testing. ACM SIGSOFT Software Engineering Notes **27**, 191–195 (2002)
9. Chen, T.Y., Ho, J.W.K., Liu, H., Xie, X.: An innovative approach for testing bioinformatics programs using metamorphic testing. BMC Bioinformatics **10**, 24 (2009)
10. Sadi, M.S., Kuo, F.-C., Ho, J.W.K., Charleston, M.A., Chen, T.Y.: Verification of phylogenetic inference programs using metamorphic testing. Journal of Bioinformatics and Computational Biology **9**, 729–747 (2011)
11. Thornton, K.: LibSequence: A C++ class library for evolutionary genetic analysis. Bioinformatics **19**, 2325–2327 (2003)
12. Excoffier, L., Lischer, H.E.L.: Arlequin suite ver 3.5: A new series of programs to perform population genetics analyses under Linux and Windows. Molecular Ecology Resources **10**, 564–567 (2010)
13. Höner, O.P., Wachter, B., East, M.L., Streich, W.J., Wilhelm, K., Burke, T., Hofer, H.: Female mate-choice drives the evolution of male-biased dispersal in a social mammal. Nature **448**, 798–801 (2007)
14. Chadeau-Hyam, M., Hoggart, C.J., O'Reilly, P.F., Whittaker, J.C., De Iorio, M., Balding, D.J.: Fregene: Simulation of realistic sequence-level data in populations and ascertained samples. BMC Bioinformatics **9**, 364 (2008)
15. Peng, B., Amos, C.: Forward-time simulation of realistic samples for genome-wide association studies. BMC Bioinformatics **11**, 1–12 (2010)
